# Dracorhodin Perchlorate Accelerates Cutaneous Wound Healing in Wistar Rats

**DOI:** 10.1155/2017/8950516

**Published:** 2017-12-03

**Authors:** Xiao-wen Jiang, Lu Qiao, Lin Liu, Bin-qing Zhang, Xue-wei Wang, Yu-wen Han, Wen-hui Yu

**Affiliations:** Department of Veterinary Medicine, Northeast Agricultural University, 59 Mucai Street, Xiangfang District, Harbin 150030, China

## Abstract

Dracorhodin perchlorate (DP) is extracted from* Dragon's blood*, which is widely used in traditional Chinese medicine, especially in wound healing. The aim of this paper is to investigate the influence of DP ointment, which contained DP dissolved in DMSO and mixed with Vaseline, on cutaneous wound healing in Wistar rats. Forty Wistar rats were divided into two groups: control and DP groups. The skin on the back of each rat was punched with two full-thickness wounds and then treated with the corresponding drug. After 3, 7, 10, 14, and 21 days, four rats were sacrificed for immunological, biochemical, and histological analyses. Compared with the control treatment, DP could significantly promote wound closure. Histological and biochemical analyses of the skin biopsies also showed that DP regulated the expression of inflammatory responses by TNF-*α* and IL-*β* and by supporting wound tissue growth and collagen deposition. Western blot revealed that* DP* could also facilitate the expression of EGF and VEGF proteins. In conclusion,* DP *promotes wound healing.

## 1. Introduction

Wound healing is the repair of damaged skin tissue which consists of three stages, inflammation, proliferation, and remodeling [1, 2]. These stages occur at a specific time, but a number of factors such as wound type, severity, wound treatment will affect all phases of wound healing, which causes delays of wound healing [3]. In clinical practice, a wet and moist condition is needed to provide an optimum healing condition, which is regarded as a better method [4]. Nowadays, many ways such as those that contain enzymes, polysaccharides of antibacterial ointment, growth factors, and other substances have been developed; a stable, lasting, and cheaper drug is an urgent problem because of the defects of these options [5].

*Dracorhodin perchlorate* is an antibacterial analogs of the red pigment anthocyanin,* dracorhodin* is known as “*Dragon's blood”* of the fruit extract of traditional Chinese drugs [6].* Dragon's blood* is well known as a traditional Chinese medicine which is processed by resin of Palm plant exudation of* daemonorops DracoBL.* Fruit spread over Java, Indonesia, Sumatra, Borneo, and so forth.* Dragon's blood* has been used by different civilizations such as the Greeks, the Romans, and the Arabs. It has several medicinal properties, such as wound healing, cicatrizant, immunomodulator, analgesic, antiulcer, antidiarrheal, antibacterial, antiviral, antihemorrhagic, anti-inflammatory, antioxidant, mutagenic and antimutagenic, antitumor, anticancer, and cytotoxic effects [7]. Some experimental studies have shown that* Dragon's blood* can promote the proliferation of fibroblasts and collagen formation and stimulate epithelium regeneration to promote wound healing.

Previous studies had demonstrated a complex effect of DP on human cells [8–12]; besides, DP has been indicated to induce apoptosis in different cell lines [6]. To identify the effective constituent in* Dragon's blood* that favors wound healing, we experimentally determined the mechanism by which DP accelerates wound healing on Wistar rat skin. But the mechanism of DP influencing cell signaling pathway remains to be fully elucidated.

## 2. Materials and Methods

### 2.1. Chemicals and Biochemicals

The following chemicals and kits were used: DP (≥99%; Pharmaceutical Inspection Institute, Harbin, China), VEGF and EGF antibodies (Sangon Biotech Institute, Shanghai, China), and TNF-*α* and IL-*β* kits (Nanjing Jiancheng Bioengineering Institute, Nanjing, China). Other reagents were of analytical grade and obtained from various commercial sources.

### 2.2. Ointment Preparation

DP (20 mg) was dissolved in DMSO (2 mL), and an aliquot of the drug mixture (20 *μ*L) was diluted to a final concentration of 200 *μ*g/mL with DMSO (980 *μ*L). The drug mixture (1 mL) was mixed with Vaseline (16 g) to prepare an ointment and then stored at 4°C.

### 2.3. Animals

Forty healthy adult male Wistar rats (Harbin Medical University in Harbin, China) weighing 250 ± 10 g were maintained under standard laboratory conditions at 22 ± 2°C and 12 h/12 h light/dark cycles and given free access to food and water.

Before the experiment was performed, the rats were subjected to adaptive breeding for 1 week and separately housed to prevent biting, which might adversely affect wound healing. Ethical approval was obtained from Northeast Agricultural University.

### 2.4. In Vivo Wound Healing Experiments

The rats were randomly divided into two groups (*n* = 20) and anesthetized by abdominally injecting chloral hydrate (10%, 0.35 mL/100 g). The skin on the back of the rats was electrically shaved and disinfected with 70% ethanol. Two full-thickness excision wounds were punched on the back of each rat by using a sterile punch biopsy (1 cm in diameter), and a tincture was applied to disinfect wounds after the excision was completed. The rats were placed in a quiet place to recover from anesthesia, and the control and DP groups received their respective treatments. Four rats from each group were euthanized on days 3, 7, 10, 14, and 21 postwounding, and the wounded areas were excised and maintained for subsequent histological and biochemical analyses.

### 2.5. Wound Healing Rate Determination

Wounds were photographed with a Canon digital camera on days 0, 3, 7, 10, 14, and 21 postwounding. The wound area was measured with a caliper for reference and Image Pro Plus (USA). The wound healing rate, which refers to the percentage of wound reduction, was calculated using the following formula: wound healing rate = (wound area on day  0 − wound area on day *n*)/(wound area on day 0) × 100%, where *n* = 0, 3, 7, 10, 14, and 21 days postwounding. Values were expressed as the percentage of wound area reduction.

### 2.6. Histological Analysis

On days 3, 7, 10, 14, and 21 postwounding, the wound tissues of the rats were collected and obliterated. The connective tissue was kept in an ice bath, fixed with 4% paraformaldehyde for 48 h, dehydrated in alcohol gradient until the tissues appeared transparent, and routinely processed under standard procedures. Tissue fragments were impregnated in paraffin at 60°C, and 5 *μ*m thick sections were cut and stained with hematoxylin and eosin (HE) and Masson's trichrome. To observe skin wound healing and collagen synthesis, we examined the prepared tissue slides and photomicrographed them with a digital camera (PowerShot G1 X Mark II camera, Canon, Inc., Japan) attached to a light microscope (Olympus Optical, Tokyo, Japan). The number of inflammatory cells and the degree of collagen deposition were analyzed using Image Pro Plus.

### 2.7. Immunohistochemistry of CD31 and Analysis of MVD

The paraffin sections were baked in a drying oven at 80°C overnight and routinely processed. Before immunohistochemical analysis was conducted, endogenous peroxidase activity was inactivated with 3% hydrogen peroxide for 10 min, and antigen was retrieved with 0.01 M citrate and EDTA buffers. Bovine serum albumin (BSA, 5%) was used to block the antigen for 20 min. The slides were incubated with rabbit anti-rat CD31 antibody (Sangon Biotech Institute, Shanghai, China) at 1 : 80 dilution and at 4°C overnight. Afterward, the slides were washed thrice with PBS, incubated again at 37°C for 30 min with secondary anti-rabbit antibody (Beyotime Biotechnology Co., Ltd., Shanghai, China) at 1 : 200 dilution, and washed thrice with PBS. Color reaction was developed with a 3,3′-diaminobenzidine-tetrachloride kit (Nanjing Jiancheng Bioengineering Institute, Nanjing, China). After the routine process was completed, the sections were observed under a microscope (Olympus BX51, Olympus Optical, Tokyo, Japan). For CD31 density counting, the sections were stained with claybank and hematoxylin and imaged under the microscope at 200x magnification.

The calculation of wound microvascular density (MVD) was based on the positive cells observed in each slide. Three low-magnification (40x) images were randomly selected according to the highest density of positive cells, and five random high-magnification (400x) images were used to count the number of positive cells. The average MVDs of the specimens were obtained.

### 2.8. Western Blot

The wounds were cut and harvested immediately in liquid nitrogen. The frozen tissues were ground with a glass homogenizer in an ice bath. Protein lysate (1 mL) and phenylmethylsulfonyl fluoride (PMSF; 1 mL) were added to a homogenizer and centrifuged at 12,000 r/min for 30 min. The supernatant was discarded to measure the protein concentration by using a bicinchoninic acid assay kit according to the manufacturer's instructions. The samples were preserved at −80°C. After the protein was preprocessed, 20 *μ*L of the protein liquid was separated onto 10% SDS-PAGE and electroblotted to enhance the chemiluminescence of nitrocellulose membranes. The membranes were incubated with a primary antibody (anti-VEGF polyclonal antibody, 1 : 500; anti-TGF-*β* polyclonal antibody, 1 : 1000; Sangon Biotech Institute, Shanghai, China) at 4°C overnight and washed thrice with TBST (TBS and 0.05% Tween). The secondary antibody (anti-rabbit IgG, 1 : 2000; Beyotime Biotechnology Co., Ltd., Shanghai, China) was then added. The membranes were further incubated at 37°C for 2 h and washed again thrice with TBST. Afterward, the membranes were analyzed with an enhanced chemiluminescent reagent. GAPDH was used as a reference and Image J (USA) software was utilized to analyze the protein expression of TGF-*β* and VEGF.

### 2.9. Cytokine Measurements

Wound tissues from the control and DP groups were homogenized using a glass homogenizer with cold PBS (0.02 M, pH 7.0–7.2), centrifuged at 5000 ×g for 5 min, and stored at −80°C until the assays were performed. Cytokines, namely, TNF-*α* and IL1-*β*, were detected through enzyme-linked immunosorbent assay (ELISA) with specific antibodies (purified and biotinylated) and cytokine standards. The experiments were carried out in accordance with the manufacturer's instructions. Optical densities at 450 nm were determined with a microplate reader (Thermo Scientific Microplate Reader, USA).

### 2.10. Statistical Analysis

Data were statistically analyzed with GraphPad (San Diego, CA, USA) and expressed as mean ± standard error of mean (SEM). Statistical variations among groups were determined through one- or two-way ANOVA followed by Tukey's posttest. *p* < 0.05 were considered significant.

## 3. Results

### 3.1. Macroscopic Aspects of DP on Wound Healing

**Figure 1:**
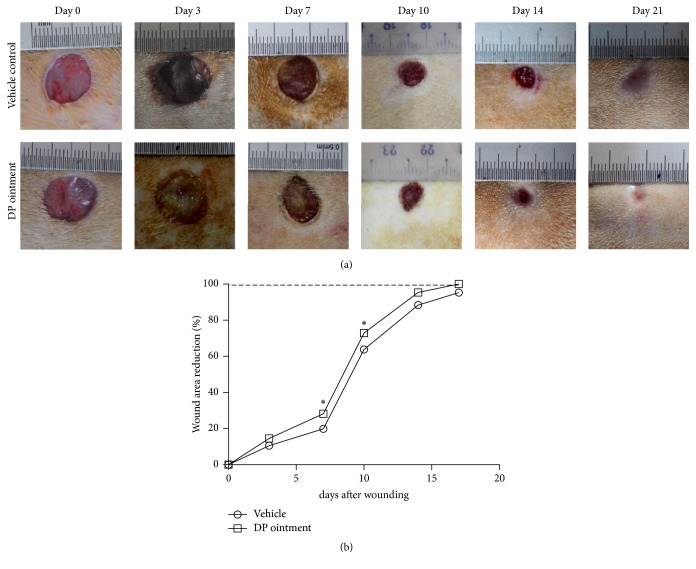
Effect of topical application of DP ointment on the wound closure and wound area reduction (wound healing rate). (a) Representative digital photographs of the full-thickness wounds of Wistar rats on days 0, 3, 7, 10, 14, and 21. (b) Wound area reduction induced by DP ointment and control ointment on days 0, 3, 7, 10, 14, and 17. Statistical data were expressed as the percentage of reduction area from the initial wound size (day 0). Values are expressed as mean ± SEM, ^*∗*^*p* < 0.05 compared with the control group.

Figures 1(a) and 1(b) show the images of wound closure after DP ointment was topically applied from days 1 to 21. DP ointment could promote the regeneration and epithelization of the wound more rapidly than the control treatment could. The wound healing rates were 14.5% and 28.2% on days 3 and 7, respectively. By comparison, these rates were 10.6% and 19.9% in the vehicle control group on days 3 and 7, respectively. After 10 and 14 days, 72.9% and 95.4% of the wound of the DP group were closed, respectively. Conversely, 63.8% and 88.4% of the wound of the control group were closed after 10 and 14 days, respectively. The speed of healing in the control group was slower than that in the DP group after 14 days, and the wounds were completely healed on day 21. The rates of the wound reduction area on day 17 were 100% and 95.3% in the DP group and the control groups, respectively. Macroscopic observation indicated that the speed of healing promoted by DP ointment was faster than that induced by the control treatment (Figure 1(a)).

### 3.2. Inhibitory Effects of DP Ointment on the Occurrence of Inflammatory Reaction

**Figure 2:**
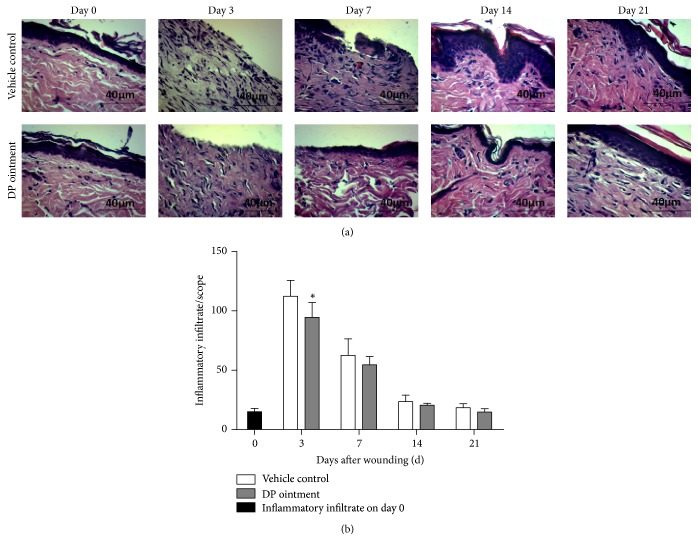
DP-treated inflammatory infiltrate at the wound site. (a) Representative HE (400x) staining of the wound sections. (b) Quantitative analysis of the inflammatory infiltrate. Image Pro Plus software was used to count the cell number in the wound tissue 3, 7, 14, and 21 days after wounding with at least five random slides per group. Values are represented as mean ± SEM (*n* ≥ 5 wounds/group), ^*∗*^*p* < 0.05 compared with the vehicle control group.

Inflammatory infiltrate recruitment was performed to evaluate inflammation by observing the HE-stained wound site. Histological analysis revealed a significant increase in the inflammatory infiltrate of the control and DP groups after 3 days (Figures 2(a) and 2(b)). A total of 112.3 and 94.5 inflammatory cells were counted in the control and DP groups, respectively. Five or more microscope slides were observed, and the number of cells was recorded statistically. Compared with that in the control group, the total number of inflammatory cells was significantly reduced in the DP group. After the total number of cells decreased on day 3, this parameter did not significantly differ between the control group and the DP group on days 7, 14, and 21. The cellular density after 14 days further reduced to normal levels in the DP group and the control group (Figure 2(b)).

### 3.3. Cytokine Detection in the Skin Wound Biopsies

**Figure 3:**
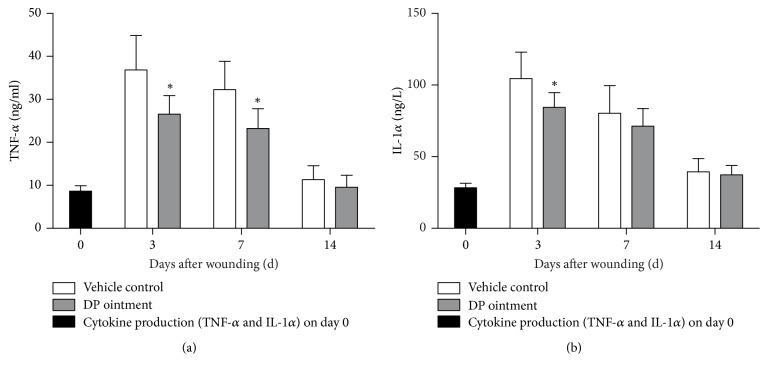
Effect of DP ointment on the cytokine production in rat skin wound tissue. Wound biopsies were obtained from the rats treated with DP ointment and control ointment on days 0, 3, 7, and 14. (a) TNF-*α* and (b) IL-1*α* were assayed through ELISA. Data are expressed as mean ± SEM, ^*∗*^*p* < 0.05 compared with the vehicle control group.

Inflammatory cells, such as polymorphonuclear cells and neutrophils, participate in skin wound healing by inducing various cytokines. Therefore, the effect of DP ointment on TNF-*α* and IL-1*α* production was investigated after wound treatment. Our results showed that DP affected the release of inflammatory cytokines. In particular, TNF-*α* levels in the tissue homogenates of the DP group significantly decreased on days 3 and 7 days (Figure 3(a)). Conversely, TNF-*α* levels in the DP group did not significantly differ from those in the control group after 7 days. IL-1*α* production was also influenced by DP treatment after 3 and 7 days. Compared with that in the control group, the IL-1*α* concentration in the DP group significantly decreased on day 3. On day 7, their differences were not significant. After 14 days, the cytokine levels did not significantly change in the control group and the DP group (Figures 3(a) and 3(b)).

### 3.4. Effect of DP Ointment Treatment on Collagen Synthesis and Wound Healing Processes

**Figure 4:**
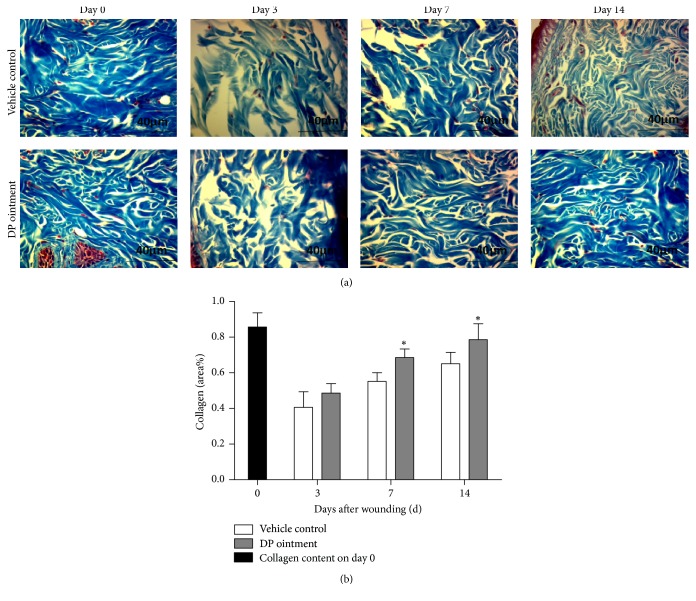
Effects of DP ointment on the collagen content in the wound tissue 0, 3, 7, and 14 days after injury. (a) Representative photomicrograph of wound sections stained with Masson's trichrome (400x). (b) Collagen content in wound tissue sections stained with Masson's trichrome as measured by digital densitometry in Image Pro Plus. Data are expressed as means ± SEM, ^*∗*^*p* < 0.05 compared with the vehicle control group.

Collagen synthesis, deposition, and degradation are important processes that occur during cutaneous wound healing. The effect of DP ointment on these processes was observed in the wound biopsies stained with Masson's trichrome and detected through computer-aided histomorphometric analyses by using Image Pro Plus to measure the collagen deposition rate (Figure 4). Computer-aided histomorphometric analyses of collagens in the wounds on day 3 showed that the density of collagen content did not significantly differ between the control group and the DP group. By contrast, the densities of collagen content of the DP group and the control group on day 7 were 53% and 68%, respectively. On day 14, their corresponding densities were 65% and 79%, and these values significantly differed between these groups (*p* < 0.05). After 14 days, the collagen level was down to normal levels in both DP and control groups.

### 3.5. Immunohistochemical Analysis of Microvascular Density (MVD)

**Figure 5:**
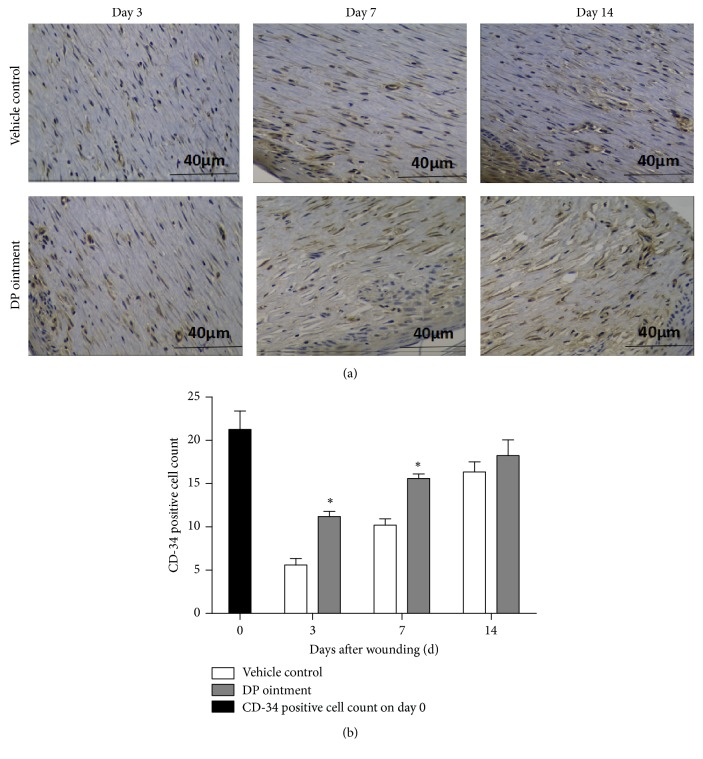
Effects of DP ointment on angiogenesis in wound tissues 0, 3, 7 and 14 days after injury. (a) Representative photomicrograph of wound sections marked with CD31 antibody and stained with 3,3′-diaminobenzidine-tetrachloride. (b) The number of CD31-positive cells in the wound. Data are represented as means ± SEM, ^*∗*^*p* < 0.05 compared with the vehicle control group.

Immunohistochemical analysis was conducted to evaluate the effect of DP on the angiogenic process of wound healing on days 3, 7, and 14 (Figure 5(a)) by using wound biopsies marked with CD31 antibody and stained with 3,3′-diaminobenzidine-tetrachloride. After detection of digital equipment analyses, MVD was determined with Image Pro Plus. The number of microvessels in the DP group was significantly higher than that in the control group after 3 days (Figure 5(b)). MVD was also influenced by DP ointment; that is, MVD was increased in the DP group after 3 days of treatment, but this increase was not significant after 7 days.

### 3.6. Effect of DP Treatment on the Protein Expression of VEGF and TGF during Wound Healing Processes

**Figure 6:**
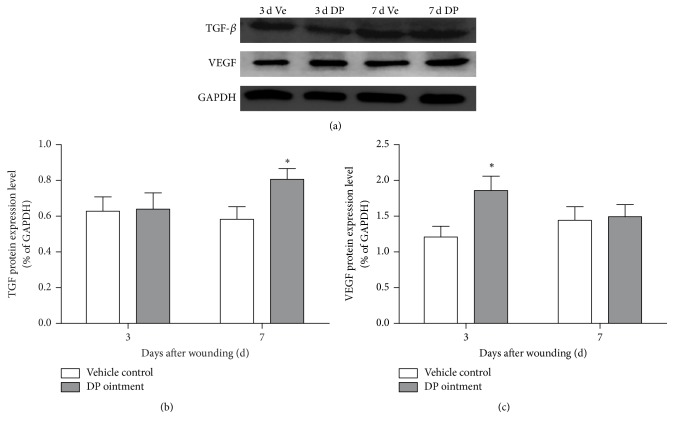
Expression of VEGF and TGF proteins in the wound tissue 3 and 7 days after injury. (a) Electrophoresis banding of wound sections. (b) Analysis of TGF-*β* and VEGF protein expression via Image J. Data are represented as means ± SEM, ^*∗*^*p* < 0.05 compared with the vehicle control group.

The secretory expression of VEGF and TGF proteins affects wound healing processes. The expression of VEGF and TGF in DP-treated wound healing was investigated in the wound biopsies through Western blot analyses. GAPDH was chosen as a reference and Image J software was used to analyze the expression of TGF-*β* and VEGF proteins (Figure 6). The expression levels of VEGF proteins on day 3 were significantly higher in the DP group than in the control group. By contrast, the difference between the DP group and the control group was not significant on day 7. The TGF protein content of the control group did not significantly differ from that of the DP group on day 3. By comparison, the difference between the control group and the DP group was significant on day 7 (*p* < 0.05). After 7 days, the expression of the two proteins did not significantly change in the control group and the DP group.

## 4. Discussion

Studies on wound healing are challenging. According to traditional Chinese medicine,* Dragon's blood* can accelerate wound healing, elicit anti-inflammatory and antioxidant effects on wound healing [13–15], and presumably speed up fibroblast proliferation and epithelial cell differentiation [16, 17]. As an important component of* Dragon's blood*, DP was used in our study to verify whether this compound contributes to wound healing. Our results revealed that the DP ointment can effectively hasten wound healing.

Inflammation plays an important role in early wound healing stages. The release and secretory of inflammatory factors such as IL-1*α*, TNF-*α*, and TGF-*β* would affect the proliferation and differentiation of fibroblast [18]. TNF-*α* was a type of polypeptide cytokines with a variety of biological activities secreted by monocyte-macrophages related to the collective immune response, acute phase response and inflammation [19]. TGF-*β* was an essential growth factor during wound healing process, which appeared immediately in the wound after release [20, 21]. The wound healing process would lag if the secretion of TGF-*β* stopped [22, 23]. In our study, the IL-1*α* and TNF-*α* levels of the DP group decreased in the early stages of wound healing. We hypothesized that a component of* Dragon's blood* possibly promotes wound healing. Some scholars have found that traditional Chinese medicine Dragon's blood had an obvious effect on wounds, ulcers, diarrhea, anticancer, and anti-inflammatory [24, 25], the effect of DP on wound healing in vitro has been rarely reported.

In wound healing, microangiogenesis plays a vital role in epithelial cell proliferation. The regeneration of blood vessels is an important index of evaluating speed wound healing [21]. VEGF is mainly through its downstream PLC*γ*1, PI3K-AKT signal pathway of endothelial cell proliferation. VEGF-A/VEGFR2 is set by PLC*γ*1 by promoting its exciting kinase c and Raf-MEK signaling pathway, eventually through the Erk1/2 pathway to promote the proliferation of vascular endothelial cells [26]. In our study, the expression of VEGF protein in the skin wounds of the DP group was higher than that in the control group on day 3. The same finding was revealed by immunohistochemical staining, but this expression did not significantly differ between the DP group and the control group on day 7. It had been reported that VEGF could accelerate the process of angiogenesis through secreting angiopoietin-2 (ANG-2) mediated by vascular endothelial cells [27]. DP can also activate the Ras/MAPK signaling pathway in human umbilical vein endothelial cells and thus enhance angiogenesis.

Collagen synthesis is essential for wound healing because collagen is an important connective tissue component, and healing requires collagen formation and deposition [28]. The quality of wound healing is also crucial during a treatment course [29]. In our study, the density of collagen content in the DP group was higher than that in the control group, and this observation indicated that collagen secretion was promoted by DP during wound healing. These results suggested that collagen synthesis and deposition in wound healing can be induced by* Dragon's blood* [25].

The active ingredients in* Dragon's blood* may play an important role in wound healing. As such, DP will be thoroughly investigated in our future studies.

## 5. Conclusion

DP can induce complete wound healing in our Wistar rat model. DP can inhibit IL-1*α* and TNF-*α* secretion to reduce inflammation and stimulate VEGF and TGF protein expression, microvessel formation, and collagen deposition to promote wound healing. Therefore, DP ointment can be topically applied to treat skin injuries.
